# Sex and Circadian Timing Modulate Oxaliplatin Hematological and Hematopoietic Toxicities

**DOI:** 10.3390/pharmaceutics14112465

**Published:** 2022-11-15

**Authors:** Sandrine Dulong, Lucas Eduardo Botelho de Souza, Jean Machowiak, Benoit Peuteman, Gaelle Duvallet, Déborah Boyenval, Elise Roth, Afag Asgarova, Yunhua Chang, Xiao-Mei Li, Adlen Foudi, Annabelle Ballesta

**Affiliations:** 1 Chronotherapy, Cancer and Transplantation, Faculty of Medecine, CNRS Campus, University of Paris-Saclay, Bat A 3rd Floor, 7 Rue Guy Moquet, 94800 Villejuif, France; 2 INSERM ATIP-Avenir and INSERM UMR-S 900, Institut Curie, MINES ParisTech CBIO, PSL Research University, 92210 Saint-Cloud, France; 3 INSERM ATIP-Avenir and INSERM UMR 1310, Faculty of Medicine, CNRS Campus, University of Paris-Saclay, 94800 Villejuif, France; 4 Hemotherapy Center of Ribeirão Preto, Ribeirão Preto 2501, SP, Brazil; 5 INSERM UMS44, 14 Avenue Paul Vaillant Couturier, 94800 Villejuif, France; 6 INSERM UMR 1310, CNRS Campus, Paris-Saclay University, 94800 Villejuif, France; 7 INSERM UMR-S 1151, Department of Immunology, Infectiology and Hematology, Institut Necker-Enfants Malades (INEM), Paris Descartes University, CNRS UMR 8253, 75730 Paris, France

**Keywords:** oxaliplatin, sex, circadian rhythms, circadian clock, chronotherapy, hematopoiesis, hematopoietic toxicity, personalized medicine

## Abstract

Oxaliplatin was nearly twice as hematotoxic, with optimal circadian timing differing by 6 h, in women as compared to men with colorectal cancers. Hence, we investigated sex- and timing-related determinants of oxaliplatin hematopoietic toxicities in mice. Body-weight loss (BWL), blood cell counts, bone marrow cellularity (BMC) and seven flow-cytometry-monitored hematopoietic progenitor populations were evaluated 72 h after oxaliplatin chronotherapy administration (5 mg/kg). In control animals, circadian rhythms of circulating white blood cells showed a peak at ZT5 in both sexes, whereas BMC was maximum at ZT20 in males and ZT13h40 in females. All BM progenitor counts presented robust rhythms with phases around ZT3h30 in females, whereas only three of them rhythmically cycled in males with a ≈ −6 h phase shift. In treated females, chronotoxicity rhythms occurred in BWL, WBC, BMC and all BM progenitors with the best timing at ZT15, ZT21, ZT15h15 and ZT14h45, respectively. In males, almost no endpoints showed circadian rhythms, BWL and WBC toxicity being minimal, albeit with a substantial drop in BM progenitors. Increasing dose (10 mg/kg) in males induced circadian rhythms in BWL and WBC but not in BM endpoints. Our results suggest complex and sex-specific clock-controlled regulation of the hematopoietic system and its response to oxaliplatin.

## 1. Introduction

Anticancer therapies may induce severe side effects on healthy organs, possibly leading to either reduced drug dosage or treatment discontinuation. These toxicities prevent patients from receiving the most effective therapies and doses and participate in pharmacological resistance. Hence, therapeutics strategies aiming to minimize toxicities while maintaining an optimal antitumor efficacy are urgently needed. In the era of precision and personalized medicine, such treatment optimization must account for determinant patient characteristics, together with physiological mechanisms at the molecular, cellular, organ and whole-body levels, which may critically impact drug response. The analysis of such detailed multi-type datasets may require the use of systems medicine approaches enabling the understanding of the complex underlying physiology through mathematical modelling [1]. Here, we aim to study the inter-related effect of sex and drug circadian timing on the toxicity of the anticancer drug oxaliplatin on several components of the hematopoietic system, towards a better handling of the drug hematological side effects.

Importantly, patient sex is a major dichotomy factor of antitumor therapy toxicities and response, as more adverse events and lower survival have generally been observed in women as compared to men receiving any type of anticancer drug [2,3,4]. The sex-specific tolerability and efficacy may be due to differences in drug pharmacokinetics (PKs) and pharmacodynamics (PDs), although more investigations are needed to decipher the precise molecular mechanisms at stake for most compounds [5]. The discovery of mechanistic explanations of sex-related therapy response may allow one to tailor not only drug doses, but also drug combinations, sequence and timing, through systems medicine approaches, towards a large benefit for the patients, especially for women [6,7].

Another therapeutic strategy that may bring substantial benefit to the patient is the account of the organism circadian rhythms [8,9]. Mammals present a circadian timing system (CTS) that controls most physiological functions throughout the day and night cycles and ensures optimal energy management. The mammalian CTS is firstly composed of a central pacemaker in the hypothalamus: the suprachiasmatic nuclei (SCN). The neurons in the SCN display endogenous circadian rhythms, where the period is exactly set to 24 h by the entrainment to external cues, such as light–dark cycles. Next, each nucleated cell of the body is endowed with a molecular clock, which spontaneously oscillates as a result of several interconnected intracellular feedback loops involving approximately 15 clock genes. The SCN send oscillating signals to these peripheral clocks though physiological cues, such as variations in body temperature or hormone levels, to ensure cell synchrony within an organ and coordinate circadian rhythms in the organism functions. As a result of this circadian organization, healthy organs may respond differently to drug exposure according to drug timing over the 24 h span, thus, leading to time-dependent levels of toxicities, as shown for more than 50 antitumor compounds [8].

Hematological toxicities are among the most frequent and severe side effects of cytotoxic drugs and may drastically decrease patient quality of life and survival [10]. Antitumor drug cytotoxicity may be observed on all hematological cell types and is one of the most common toxicities leading to treatment discontinuation [11]. Furthermore, myelosuppression-related death may account for up to 1.3% of treated patient death [12,13]. The hematopoietic cells within the bone marrow are organized in a deep hierarchy, with the hematopoietic stem cell (HSC) at the apex followed by a myriad of downstream progenitors with different degrees of lineage commitment and proliferation [14]. Immature bone marrow progenitors ultimately differentiate into mature blood cells that reach the systemic circulation. These populations present in the bone marrow can nowadays be identified and quantified by flow cytometry using a combination of cell surface markers [14,15]. 

The hematopoietic system is under the tight control of the CTS [16,17]. Although circadian rhythms of the hematopoietic system in humans were first described in the 50 s with the evidence of variations in circulating blood cell counts, the study of the circadian organization of the bone marrow is more scarce and more recent. To the best of our knowledge, only two preclinical studies investigated the daily variations in hematopoietic immature populations in the bone marrow [17,18]. The complex structure of the hematopoietic system and its time dependency may largely impact on the appearance of drug hematological toxicities. First, cell-type-specific drug sensitivity may be observed according to hematopoietic cell maturity, along which genes involved in key intracellular processes may be alternatively turned on and off. As an example, temozolomide, the cornerstone of brain tumor chemotherapies, was highly toxic for human monocyte, while dendritic cells and macrophages were resistant to the drug though re-activation of key DNA repair pathways [19]. Next, the circadian variations in the hematopoietic system logically translate into different tolerability to many anticancer drugs depending on administration timing [8].

This article focuses on the anticancer drug oxaliplatin, which is widely employed in combination with 5-fluorouracil and irinotecan for the treatment of gastro-intestinal cancers [20,21]. Such drug combinations are responsible for severe hematological toxicities both in mice and in patients, among other types of side effects, in a timing-dependent manner [22,23]. Sex has been identified as a key factor of tolerability for these combination chemotherapies. In patients receiving oxaliplatin in combination with irinotecan, female sex and age over 70 years were significantly associated with higher incidence of severe hematological and digestive toxicity, with no differences in treatment efficacy [24]. Moreover, being female was a risk factor for severe leukopenia incidence in irinotecan-treated patients [12]. Further, sex was a strong risk factor for toxicity in patients receiving 5-fluorouracil, showing the importance of evaluating male and female patients separately [25]. There are also interactions between sex and circadian timing, as shown for irinotecan monotherapy in mice and for the 5-fluorouracil/oxaliplatin and 5-fluorouracil/oxaliplatin/irinotecan in patients [26,27,28,29]. The chronomodulated administration of oxaliplatin (peak at 4 p.m.) and 5-fluorouracil (peak at 4 a.m.) increased the survival of male patients but decreased that of female metastatic colorectal cancer patients, as compared to non-circadian-based administration of the same drug doses [28]. More recently, optimal timing of irinotecan administered in combination with 5-fluorouracil and oxaliplatin was found to occur in the early morning for men and in the early afternoon for women patients, with larger amplitudes for women [29]. 

Regarding oxaliplatin preclinical investigations, chronotoxicity studies were performed in male mice and hematological and intestinal toxicities displayed circadian rhythms with the worst timing around ZT7 (Zeitgeber Time; mouse subjected to cycles of 12 h of light/12 h of darkness, ZT0 defining light onset [30,31]). However, nothing is known about the cellular determinants of oxaliplatin toxicities on the hematopoietic system and the implication of sex on them. The present study aimed to evidence the sex-specific circadian organization of the hematopoietic system and its response to the anticancer drug oxaliplatin, depending on sex, drug dosing and timing. Starting at a physiological dose of 5 mg/kg administered at six circadian times, oxaliplatin activity on body weight, circulating blood cell counts, bone marrow cellularity and progenitor composition was investigated in male and female mice. Next, the effect of increasing oxaliplatin dose to 10 mg/kg on drug hematological chronotoxicity was studied in male mice across the 24 h span.

## 2. Materials and Methods

**Figure 1:**
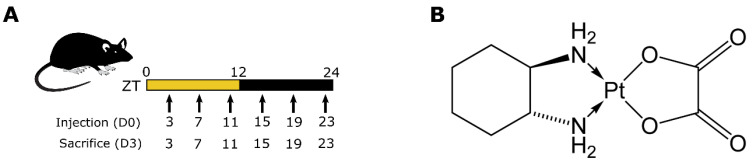
Experimental Methods: (**A**) Mouse investigation protocol for circadian drug injection and animal sacrifice. (**B**) Oxaliplatin molecular structure.

**Animals and synchronization.** The studies were carried out in male and female B6D2F1 mice, 7 weeks of age (Janvier, Le Genest-Saint-Isle, France). Mice were synchronized using facilities dedicated to circadian studies, with an alternation of 12 h of light (L, 300 lux) and 12 h of darkness (D) (LD 12:12), with food and water ad libitum for 3 weeks prior to any intervention (Figure 1A). Zeitgeber Time 0 (ZT0) and ZT12 corresponded to L onset and D onset, respectively. All manipulations during the dark span were performed under dim red light (<7 lux).

**Experimental design and tissue collection.** Clinical oxaliplatin (OHP) (Figure 1B) solution (Eloxatin, Sanofi-Aventis, France) was diluted in sterile water daily prior to injections. The final drug solution, with concentration 10 mg/mL, was injected intravenously. A single dose of oxaliplatin was administered at ZT3, ZT7, ZT11, ZT15, ZT19 or ZT23 at 0 (vehicle only) or 5 mg/kg of body weight for female mice (8 animals/ZT/dose) and 0, 5 and 10 mg/kg for male mice (8 animals/ZT/dose, Figure 1A). After injection, mice survival and BWL were evaluated daily for 72 h. Mice were sacrificed 72 h after injection when the toxicities were assumed to be the highest, as suggested by previous studies [30,31]. Each group of mice was sacrificed at the same ZT as that of injection. Blood and bone marrow samples were collected as described below.

**Blood cell count.** Blood samples were collected in heparinized tubes and kept rocking until analysis. Blood cell counts were obtained using the veterinary hematology analyser VetScanHM5c™ (Abaxis Europe, Griesheim, Germany).

**Bone marrow collection and cellularities.** Bone marrow was collected from both hind limbs of each mouse, as follows. Bones were flushed with sterile phosphate-buffer saline (PBS, Roche, France) containing 2% of heat-inactivated fetal bovine serum (FBS, Fisher Scientific) several times to avoid cell clumps. Cells were centrifuged at 300× *g* for 5 min and pellets were resuspended in cold RBC lysis buffer (Roche, France) and incubated on ice for 4 min. After addition of PBS, to stop RBC lysis, cells were filtered through a 40 μm cell strainer to obtain a single cell suspension. Cells were centrifuged again at 300× *g* during 5 min and pelleted in PBS for scoring. Cells were then scored manually in triplicates using a Malassez hemocytometer to obtain cellularity values. Any datapoint below the value of 20 × 10^6^ cells in the control conditions were filtered out as such unrealistically low levels were likely to be due to technical variability (11 excluded datapoints out of 96).

**Figure 2:**
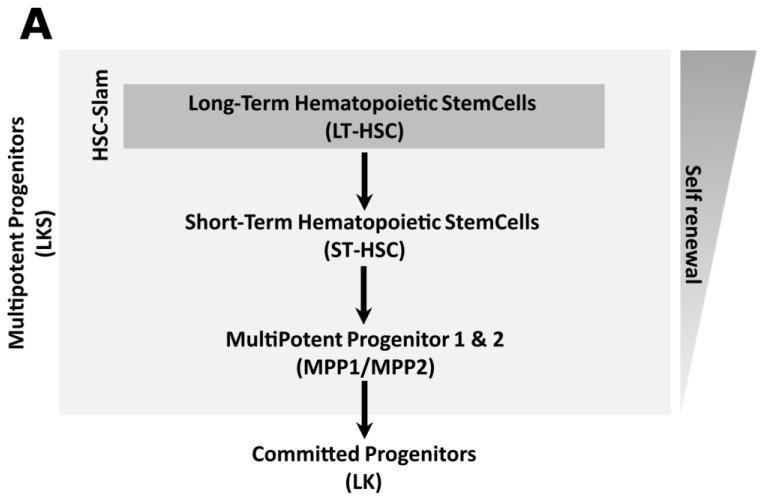

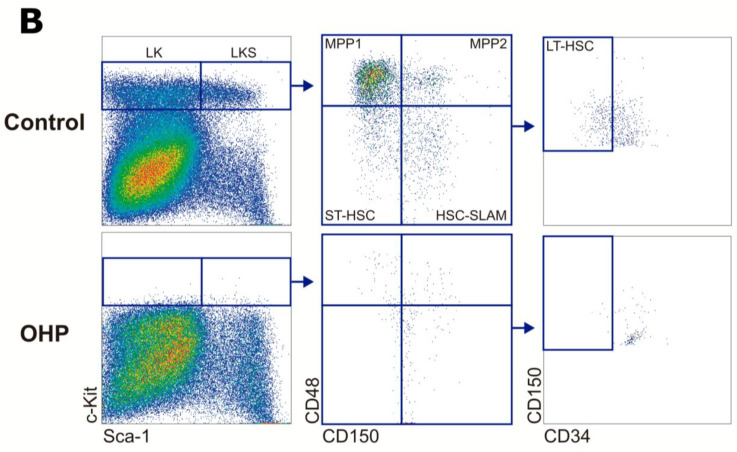
(**A**) Hierarchy/heterogeneity of the hematopoietic immature cell populations investigated in this study. (**B**) Gating strategy and hierarchical relationship of the hematopoietic populations of interest. Representative flow cytometry plots of a control (**upper panels**) and OHP-treated mouse (**lower panels**). After exclusion of debris and cell doublets, cells were gated on Lin-PI- population. Lin- viable cells were then used to investigate seven progenitor populations.

**Flow cytometry to study bone marrow hematopoietic progenitors.** A modified FACS ARIA flow cytometer with five lasers (UV 300 nm, violet 405 nm, blue 488 nm, green 532 nm, red 633 nm) was used for analysis and sorting of hematopoietic progenitor cells. Immunostains were performed as described before [32]. The following antibodies (BD Biosciences) were used for staining: PE-Cy5-conjugated lineage (Lin) markers CD3ε (145-2C11), B220 (RA3-6B2), Gr1 (RB6-8C5), Mac1 (M1/70) and Ter119, CD117/c-Kit (2B8, APC-Cy7), Sca-1 (D7, BV421), CD48 (HM48.1, APC), CD150 (TC15-12F12.2, PE) and CD34 (RAM34, FITC). Propidium iodide (PI) was used to exclude dead cells. Data were analyzed using the FlowJo 10.7.1 software (BD Biosciences). For population analysis, debris was excluded based on SSC/FSC plots, followed by doublets exclusion and gating on Lin^-^PI^-^ cells. Bone marrow progenitors were quantified by flow cytometry using a combination of cell surface markers as follows (Figure 2A,B). The strategy used to analyze the mouse hematopoietic system consisted of excluding cells expressing at least one lineage-specific marker (e.g., Ter119, B220, CD3, Mac1, Gr1) followed by an initial segregation between committed myeloid progenitors (Lin^-^c-Kit^+^Sca-1^−^, “LK” cells) and hematopoietic stem/progenitor cells (Lin^-^c-Kit^+^Sca-1^+^; “LKS” cells). Based on the expression of the SLAM markers CD48 and CD150 [14], LKS cells could be further divided into early progenitors referred to as short-term (ST)-HSCs (Lin^−^c-Kit^+^Sca-1^+^CD48^−^CD150^−^), MPP1 (Lin^−^c-Kit^+^Sca-1^+^CD48^+^CD150^−^) and MPP2 (Lin^−^c-Kit^+^Sca-1^+^CD48^+^CD150^+^) as well as a population containing HSC, but still heterogeneous, referred to as HSC-SLAM (Lin^−^c-Kit^+^Sca-1^+^CD48^-^CD150^+^). Such notation was chosen for the sake of clarity, although ST-HSCs may be considered as SLAM cells as well. Finally, expression of CD34 could be used to identify HSC with long-term repopulation capacity (LT-HSCs) (Lin^−^c-Kit^+^Sca-1^+^CD48^−^CD150^+^CD34^−^) [15]. The gating strategy to discriminate all populations of hematopoietic stem/progenitor cells is shown in Figure 2.

**Statistical analysis.** To measure the drug effect, we computed circulating blood cells, bone marrow cellularity and progenitor counts in treated conditions as a percentage of their values in the control conditions as:

$$Treated\_relative\left(ZT\right)=\frac{\mathrm{Treated}\left(\mathrm{ZT}\right)}{\mathrm{Control}\left(\mathrm{ZT}\right)}*100$$

These metrics evaluate the relative change in the observed variable in treated conditions as compared to control. The least toxicity is achieved when this quantity is close to 100%.

The effect of sex, circadian timing and oxaliplatin doses on survival, body-weight loss, cell counts and percentages were tested though multi-factor ANOVA (PASW, IBM). The statistical significance of circadian rhythms was determined with cosinor analysis, programmed in Matlab (MathWorks, US). This routine fits a 24 h cosine curve of the form:

$$f\left(t\right)=M+Acos\left(\frac{2\pi }{24}\left(t-\varphi \right)\right)$$

where t is the time expressed in hours, M is the mesor (rhythm-adjusted mean), A the amplitude (half the difference between curve minimum and maximum) and φ the phase (time of maximum). To compare cosines of different mesors, it is also convenient to compute the relative amplitude $\frac{A}{M}$. The superiority of the best-fit cosine is compared through an F-test. The threshold of statistical significance for ANOVA and cosinor was set to *p* < 0.05.

## 3. Results

### 3.1. Circadian Rhythms of Hematopoietic Components under Control Conditions

As a start, we studied the circadian variations in several types of blood and bone marrow cells in mice receiving an injection of sterile water, which was expected to minimally alter physiological conditions. Outputs were monitored 72 h after a single injection of vehicle in male and female mice, at six circadian times.

#### 3.1.1. Circulating Blood Cells

**Figure 3:**
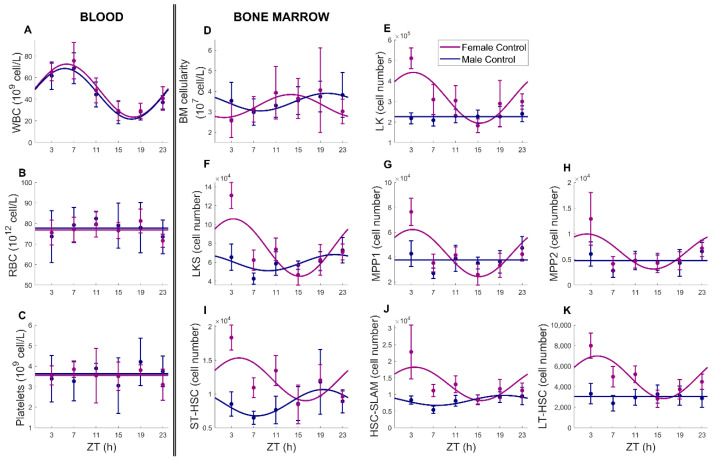
Sex- and timing-dependent blood and bone marrow cell counts in control animals (**A**–**C**) Circulating blood cells, (**D**) bone marrow cellularity (**E**–**K**) bone marrow immature progenitors. Dots represent raw data (mean ± SD) and solid lines are the best-fit cosinor curves.

Significant differences pending circadian time were observed for WBC, lymphocytes, monocytes and neutrophils (ANOVA *p* < 0.001, cosinor analysis, Figure 3A–C and Figure S1, Table 1 and Table S1) and were identical between males and females (ANOVA, *p* ˃ 0.05). Accordingly, similar mesors, amplitudes and acrophases were found in males and females with time of maximum counts around ZT 5 (Table 1). Circulating WBC were mainly lymphocytes, which accounted for 83% and 88% of WBC mesor in males and females, respectively. As a consequence of lymphocyte predominance, cosinor parameters of WBC were close to that of lymphocytes. In both males and females, acrophases of all types of WBC were aligned. Regarding RBC and platelets, they did not display circadian variations, either in males or females (ANOVA and cosinor *p* > 0.05, Figure S2).

#### 3.1.2. Bone Marrow Cellularity

Variations in bone marrow cell counts were not statistically validated by ANOVA, between males and female or pending on the time of administration (*p* ˃ 0.05) in the analysis of untreated animals (Figure 3D). However, the cosinor test concluded with significant circadian rhythms in bone marrow cellularity in both sexes with similar mesor and moderate amplitude. We could observe sex-related differences in the phases that were equal to ZT20h10 ± 1 h 28 in males and ZT13h42 ± 1 h 23 in females. The phase shift observed between circulating and bone-marrow-located WBC was equal to 14 h 49 ± 1 h 53 and 8 h ± 1 h 47 in males and females, respectively, so that both quantities displayed rhythms in antiphase, as expected from the literature (Table 1, [33,34]).

#### 3.1.3. Bone Marrow Hematopoietic Progenitors

The circadian rhythms of seven hematopoietic progenitor populations of different maturity were investigated in the bone marrow: LK, LKS, MPP1, MPP2, ST-HSC, HSC Slam and LT-HSC (see Methods). Relative percentages of these cell populations over all myeloid progenitors, defined as Lin^−^c-Kit^+^, were similar in both sexes. Indeed, committed myeloid progenitors (LK cells) represented 79% of all myeloid progenitors in males and 81% in females, whereas stem cells or uncommitted progenitors (LKS) accounted for 21% in males and 19% in females. Next, LKS cells could be further separated, using CD48 and CD150 SLAM markers, into early progenitors referred to as short-term (ST)-HSCs (14,6% of all LKS cells in males and 16% in females), MPP1 (63% in males and 57% in females), MPP2 (8% in males and 8.7% in females) and HSC-SLAM (13.8% and 17.5% in females). Finally, HSC with long-term repopulation capacity (LT-HSCs) represented about 37% of all HSC-SLAM cells in both sexes (Table 1).

Differences between male and female under control conditions in LK, LKS, ST-HSC, HSC Slam and LT-HSC counts were statistically significant (ANOVA *p* < 0.001). Concerning females, all progenitor populations displayed statistically significant circadian rhythms (ANOVA and cosinor *p* < 0.05, Table 1). For male mice, only LKS, HSC-Slam and ST-HSC displayed circadian variations (cosinor *p* < 0.05, Figure 3). We observed significantly higher relative amplitudes in females than in males, with an average across all progenitor populations of 29% ± 7.5% of the mesor and 18% ± 6% of the mesor, respectively. The acrophases of progenitor types were clustered between ZT19h14 and ZT23h09 in males and between ZT3h04 and ZT5h16 in females (Figure 3E–K, Table 1).

### 3.2. Timing-Dependent Oxaliplatin Toxicities in Male and Female Mice Treated at Equidose (5 mg/kg)

Next, body-weight loss, circulating blood cells counts, bone marrow cellularity and progenitor counts were monitored 72 h after a single injection of vehicle (control) or oxaliplatin (5 mg/kg) in males and females, at six times of administration. The intention was to test drug dose ranges equivalent to doses used in the clinics to ensure the translational value of our findings. Oxaliplatin maximum plasma concentrations (C_max) in cancer patients receiving 130 mg/m^2^ are reached within a few minutes after injection and do not exceed 4 mg/L, for any infusion duration [35,36]. Boughattas et al. treated mice with 17 mg/kg, a highly toxic dose, which induced a plasma C_max of 16 mg/L, reached in the first few minutes after intravenous injection, which is around four-fold higher than maximum plasma drug concentrations in patients [30,31]. Assuming a linear relationship between dose and initial plasma levels, we, thus, chose to divide the dose of 17 mg/kg by approximately 4 and selected a physiological dose of 5 mg/kg for this study.

#### 3.2.1. Survival and Body-Weight Loss

**Figure 4:**
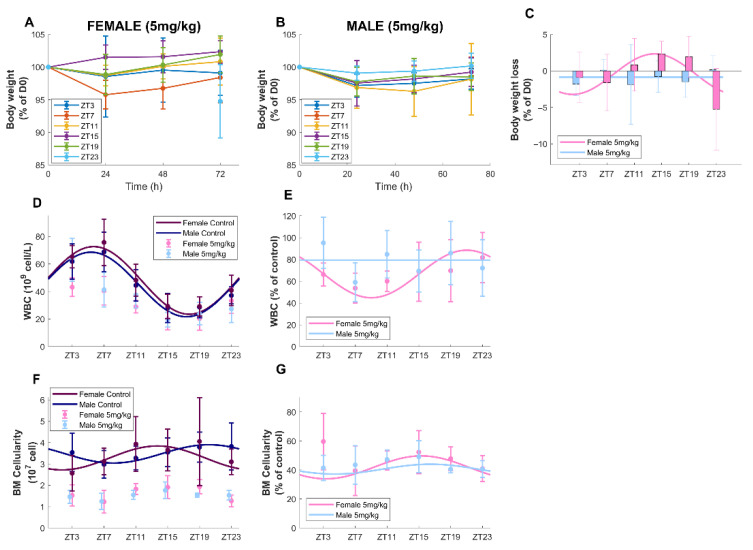
Sex- and timing-dependent body-weight loss (BWL), circulating white blood cells, bone marrow cellularity counts after oxaliplatin injection. (**A**,**B**) BWL over the 72 h following a single administration of oxaliplatin at indicated dose and times of administration (ZT). (**C**) Circadian rhythms of BWL 72 h after oxaliplatin injection, raw data (mean ± SD) and best-fit cosinor (curves). (**D**,**E**) WBC and (**F**,**G**) bone marrow cellularity. Panels (**D**,**F**) show cell concentrations in control and treated datapoints (mean ± SD) conditions. Panels (**E**,**G**) show ratios between treated and control conditions (datapoints, mean ± SD). On all panels, solid lines are the best-fit significant cosine curves.

No mortality was found in both sexes, administered 5 mg/kg of oxaliplatin, at any circadian timing. During the 72 h following the injection, average individual body-weight loss (BWL, expressed in % of weight at day 0) was equal to −0.44% ± 0.6% (mean ±SD) for the female mice and −1.16% ± 0.45% for males (Figure 4A–C). The difference according to sex was statistically validated (ANOVA, *p* = 0.029), as well as that according to ZT of injection (ANOVA, *p* = 0.027) and their joint effect (*p* = 0.001). Next, cosinor analysis was performed on BWL measured 72 h after the start of the treatment. It showed significant rhythms in the BWL of female mice treated at 5 mg/kg (*p* = 0.0079) but not in that of males treated at the same dose (*p* = 0.49). The best time of oxaliplatin administration for females was ZT13h59 ± 1h10 and absolute amplitude was 2.88% ± 3.89% of initial BW (Figure 4A,C).

#### 3.2.2. Circulating Blood Cell Counts

When considering both treated and untreated conditions, there were no sex-related dependencies in total WBC, lymphocytes, RBC or platelet counts, but statistically significant differences according to sex for monocytes and neutrophils (ANOVA *p*< 0.003). In treated conditions, on average, for all times of administration, oxaliplatin decreased the mean level of all WBC types but not monocytes, compared to control conditions for both sexes (ANOVA *p* < 0.014, Figure 4D,E and Figure S1, Table 1 and Table S1). WBC mean level was decreased upon oxaliplatin injection by 19.13% in males and by 35.28% in females.

Administration timing had a statistically significant impact on counts of all WBC types (ANOVA *p* < 0.022). Oxaliplatin chronotoxicity was further assessed by computing the difference between circulating cell numbers in control and treated animals for each ZT (Figure 4D,E and Figure S1). No rhythms of toxicity were observed for RBC and platelets (cosinor *p* > 0.05, Figure S2). A circadian rhythm in oxaliplatin toxicity on WBC was validated in females (cosinor, *p* = 0.004), but not in males (Table 1). For females, the best time of administration was around ZT21, which led to a decrease of ≈25% of WBC count from normal conditions, as compared to a decrease of ≈40% at worst timing. A closer look at oxaliplatin toxicity on the different types of WBC revealed that chronotoxicity rhythms were only observed for lymphocytes in females (cosinor *p* = 0.0013, Figure S1, Table S1).

#### 3.2.3. Bone Marrow Cellularity

Oxaliplatin administration drastically reduced bone marrow cellularity, similarly in males and females, in a timing-specific manner (ANOVA, *p* < 0.001). On average, over the 24 h window, a decrease of 59% in bone marrow cellularity compared to control was observed for males and females (Figure 4F,G). Chronotoxicity rhythms were found for both sexes (cosinor *p* < 0.02) with best time of administration at ZT15h19 ± 1 for females and ZT16h ± 1 h 26 min for males (Figure 4F,G, Table 1).

#### 3.2.4. Bone Marrow Hematopoietic Progenitors

**Figure 5:**
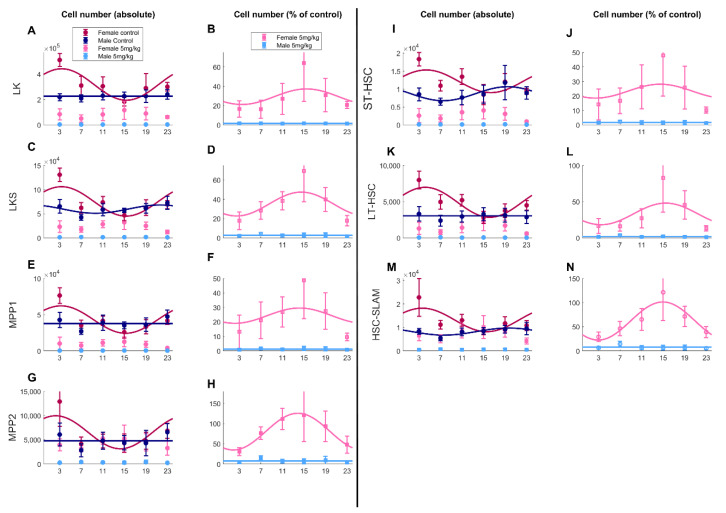
Sex and timing-dependent bone marrow progenitor cell numbers for the seven studied populations. Panels (**A**,**C**,**E**,**G**,**I**,**K**,**M**) show cell numbers in control (datapoints, mean ± SD) and treated (bars) conditions. Panels (**B**,**D**,**F**,**H**,**J**,**L**,**N**) show ratios between treated and control conditions (datapoints, mean ± SD). On all panels, solid lines are the best-fit significant cosine curves.

Seven hematopoietic progenitor populations of different maturity were investigated: LK, LKS, MPP1, MPP2, ST-HSC, LT-HSC and HSC Slam (see Section 2). Treatment, sex and treatment*sex had a significant impact on percentages of all cell types (ANOVA, *p* < 0.001). For all bone marrow cell types, higher toxicities were observed in males than in females with an average decrease in population percentages due to oxaliplatin as compared to control conditions of more than 92% in males and of 57% in females (Figure 5, Table 1). Concerning cell-type-specific drug response, we observed inter-cell population variability with greater toxicities of LK, MPP1 and ST-HSC in both males and females (decrease of 96, 97 and 96%, respectively, in males and 83, 71 and 73% in females). Lowest toxicities were observed in MPP2 and HSC-Slam (decrease of 84 and 82% in males and only 4.7 and 29% in females). Oxaliplatin chronotoxicity was assessed by computing the difference between control and treated animals for each ZT (Figure 5). A circadian rhythm of the drug toxicity was validated on all cell types in females (cosinor, *p* < 0.001) and solely for LT-HSC in males (Table 1). Relative amplitudes were consistently greater in female mice as compared to males. Interestingly, phases were shifted by +3 h to +6 h from males to females (Figure 5).

### 3.3. Circadian Time-Dependent Oxaliplatin Toxicities in Males upon Dose Increase

Here, we compared the effect of a single injection of oxaliplatin (5 mg/kg or 10 mg/kg) in males at six times of administration 72 h after injection on body-weight loss, circulating blood cells counts, bone marrow cellularity and progenitors counts.

#### 3.3.1. Survival and Body-Weight Loss

**Figure 6:**
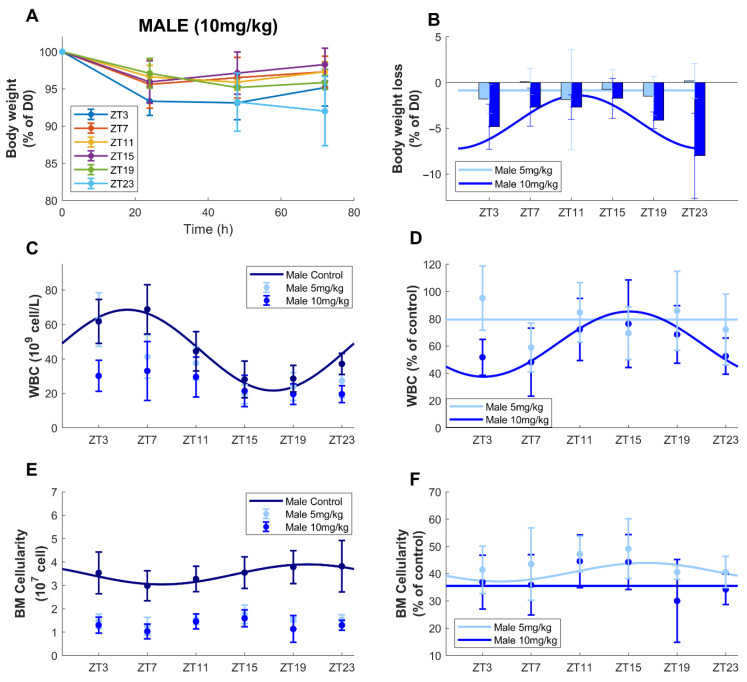
Timing-dependent body-weight loss (BWL), circulating blood cells, bone marrow cellularity counts after oxaliplatin single injection. (**A**) BWL over the 72 h following a single administration of oxaliplatin at indicated dose and times of administration (ZT). (**B**) Circadian rhythms of BWL 72 h after oxaliplatin injection, raw data (mean ± SD) and best fit cosinor (curves). (**C**,**D**) WBC and (**E**,**F**) bone marrow cellularity. Panels C and E show cell concentrations in control and treated datapoints (mean ± SD) conditions. Panels D and F show ratios between treated and control conditions (datapoints, mean ± SD). On all panels, solid lines are the best-fit significant cosine curves.

As for the 5 mg/kg dose, no mortality was found in male mice receiving 10 mg/kg at any circadian timing. Average BWL 72 h after treatment, expressed in % of weight at day 0, was equal to −4.02 % ± 0.41 %, which was more than three-fold greater than that found after a 5 mg/kg injection (Figure 6A,B). Statistical differences appeared according to the dose (ANOVA, *p* = 0.0001). BWL variations were also significatively impacted by the circadian time of treatment (ZT) as a single factor (*p* = 0.014) or combined with dose (*p* = 0.001). Cosinor analysis revealed significant circadian rhythms in BWL 72 h after treatment (*p* = 0.0004), contrary to results obtained in males treated at 5 mg/kg. The best times of oxaliplatin administration were ZT11h41 ± 48 min and the amplitude was 2.58 ± 0.64% of initial BW.

#### 3.3.2. Circulating Blood Cell Counts

When taking together all treated (5 and 10 mg/kg) and untreated male mice, there were statistically significant differences according to the dose for all blood cell types, except for monocytes (ANOVA *p* < 0.004). For all times of administration, oxaliplatin (10 mg/kg) decreased the mean level of all blood cell types, compared to control conditions, WBC being decreased by 41.72% (*p* < 0.001, Figure 6C,D and Table 1). A circadian rhythm of the difference between circulating WBC in control and treated mice (10 mg/kg) was found (*p* = 0.0005), contrary to the results in males treated at 5 mg/kg. The best time of administration was ZT15h06 ± 1h15 and the relative amplitude was equal to 38.87 ± 19.53% of mesor. Regarding the different types of WBC, chronotoxicity rhythms were only observed for lymphocytes and neutrophils in males (10 mg/kg) (cosinor, *p* = 0.0008, *p* = 0.025, Figure S1, Table S1). As for the dose of 5 mg/kg, no rhythms of toxicity were observed for RBC and platelets (ANOVA and cosinor *p* > 0.05, Figure S2). 

#### 3.3.3. Bone Marrow Cellularity

Oxaliplatin administration drastically reduced bone marrow cellularity in a dose-specific manner in male mice (ANOVA, *p* < 0.001). On average, over the 24 h window, a decrease of 64% for males treated at 10 mg/kg compared to control was observed, as compared to 59% for males treated at 5 mg/kg (Figure 6). The differences between the cellularity in treated animals and the average cellularity in control mice did not display circadian variations (Figure 6E,F, Table 1).

#### 3.3.4. Bone Marrow Hematopoietic Progenitors

Dosage had a significant impact on percentages of all cell types (LK, LKS, MPP1, MPP2, ST-HSC, LT-HSC and HSC) (ANOVA, *p* < 0.0001) as higher toxicities were observed at the dose of 10 mg/kg as compared to 5 mg/kg (Figure S3, Table 1). Circadian rhythms were not observed for the drug toxicity on any cell type (cosinor *p* > 0.05).

## 4. Discussion

The dependency of oxaliplatin toxicities on both patient’s sex and on circadian timing of administration appealed for an exploration of underlying physiological mechanisms towards explanations of such phenomena at the organ and cell population level. Here, we investigated sex-specific circadian rhythms of key components of the hematopoietic system, together with their response to oxaliplatin chronotherapy in male and female mice. Circadian studies of oxaliplatin tolerability have been previously performed on male mice, but similar data in females were missing [31]. Thus, this investigation contributes to balancing the bias of using only male mice in experiments as the US government has urged the research community to do [2]. Next, another innovative aspect of this study lies in the use of multi-flow cytometry to investigate cell-type-specific drug response of bone marrow progenitors of different maturities. To our knowledge, this is the first time that circadian rhythms of seven populations of bone marrow progenitors and their oxaliplatin sensitivity are reported with respect to sex and drug dosing.

**Figure 7:**
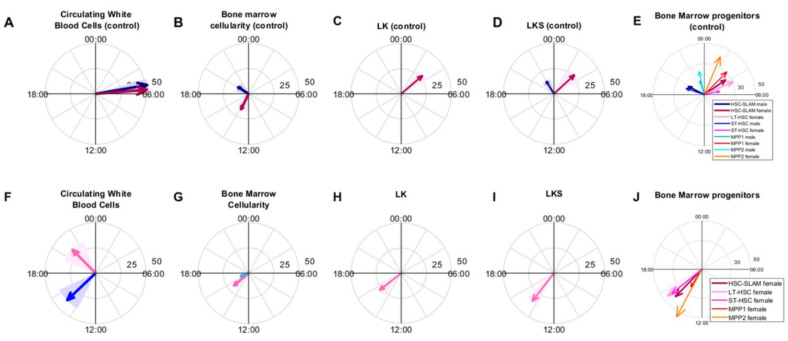
Polar plots of circadian rhythms in key components of the hematopoietic system in control conditions (**A**–**E**) and under oxaliplatin treatment (**F**–**J**). Blue colors refer to males and pink colors to females.

Results in the absence of drug allowed us to describe the normal circadian physiology of hematopoietic components, in a sex-specific manner (Figure 7A–E). We observed a circadian rhythm of the total circulating WBC count, which was similar in males and females, as shown previously [37]. Its peak was located around ZT5h30 in control animals with a large amplitude, close to 50% of the mesor, which agreed with previous observations [16,17,31,37,38]. In particular, Stenzinger et al. found circadian rhythms in circulating WBC in blood and spleen but not in bone marrow with an acrophase between ZT2 and ZT5, in agreement with our data [17]. Moreover, they showed that an attenuation or even a disappearance of these rhythms was observed in clock gene Bmal1^−/−^ animals, demonstrating that these time variations originated from a control of the circadian clock [17]. Next, we showed that, in untreated animals, BM cellularity displayed circadian variations in moderate amplitude, with a peak at ZT20h10 in males and a phase shift of +17 h between males and females. In both sexes, BM cellularity rhythms were in antiphase with that of circulating WBC, as documented in the literature, which may reflect cell trafficking throughout the day and night cycles [33,34]. Finally, all BM progenitor counts presented robust rhythms with aligned phases in females, whereas only three out of seven populations were rhythmic in males with lower amplitudes. Regarding the phases, they were clustered in a 3 to 4 h window between ZT2h16 and ZT4h22 for females and between ZT19h40 and ZT21h39 for males, leading to a phase shift of +6 to +9 h between bone marrow circadian physiology in males and females. Several publications concluded that the regulation of hematopoietic stem or progenitor cells (HSPCs) was controlled by the circadian clock under steady-state conditions [39]. Both HSPC mobilization into the blood stream and their blood counts displayed circadian variations in human or mouse studies, with highest blood HSC levels in the resting phase [17,18,39,40]. However, those studies were based on blood samples and, to our knowledge, our results constitute the first documentation of circadian rhythms of HSPCs in the bone marrow. Mendez Ferrer et al. showed daily rhythms in LKS cell counts in blood with a peak at ZT 5 in male C57Bl6J mice [18]. As circadian rhythms in the bone marrow are generally in phase opposition to those observed in the blood, these data tend to align to our results on LKS circadian counts, peaking at ZT21h39 ± 1 h 14 in male B6D2F1 mice. Overall, although circadian rhythms of circulating blood cells were similar in males and females, the upstream cell dynamics in the bone marrow presented marked sex-related differences with higher amplitudes in females and a male-to-female phase shift of more than a quarter of a 24 h period.

Next, male and female mice were treated with a single injection of oxaliplatin at a dose of 5 mg/kg at six circadian times of administration (Figure 7F–J). This treatment did not cause any mortality or severe BWL up to 72 h after the injection, for any conditions. Regarding blood toxicities, as RBC and platelets have longer half-lives than WBC, the period of 72 h after treatment was not long enough to reveal any toxicity that oxaliplatin could have had on their progenitors by solely observing circulating cells. Indeed, oxaliplatin targets dividing cells so that its influence on circulating blood cell numbers may be primarily due to its cytotoxicity on their progenitors. At 5 mg/kg, males displayed almost no BWL and moderate hematotoxicity on circulating WBC (decrease of 21% with respect to control on average), but a large decrease in BMC of 60% as compared to control and an almost complete depletion of all immature cell populations of 92%. Almost no chronotoxicity rhythms were found apart from that of the BMC with a weak amplitude of 8% of the mesor and a best timing at ZT16 and that of LT-HSC progenitors, with a negligible amplitude of 0.8% of the mesor and best timing at ZT8 (Table 1). Indeed, BWL and WBC toxicity were relatively low, which may explain the absence of timing-dependent variations in these endpoints. Conversely, large BM toxicities may explain the lack of circadian rhythms in BM endpoints in males. Taken together, these findings advocate for the study of more long-term consequences of BM depletion due to oxaliplatin exposure in males. Next, females administered with oxaliplatin at 5 mg/kg displayed timing-dependent BWL—which presented a mesor close to that of males treated at the same dose, more pronounced toxicity on WBC (decrease of 34% with respect to control on average) and equivalently high toxicity at the level of the bone marrow cellularity. A decrease in BM progenitor populations of more than 50% was observed in females, which was less drastic than in males. This sex specificity in bone marrow drug sensitivity suggested that the long-term hematopoietic cell renewal process might be more affected by oxaliplatin exposure in males than in females. Of note, Nakada and colleagues reported that BM cells from female mice proliferated more than that of similarly aged males, although no significant differences were observed in the BM composition at the steady state [41]. That included not only HSC-SLAM cells but also all MPP subsets [41]. This phenomenon coincides with previous studies, which attempted to study the cell cycle of BM cells using either genetically modified cells [42] or chemicals [43,44]. Interestingly, we could note that the toxicity of oxaliplatin on the MPP2 cell population was very low in female mice (decrease of 4.7% as compared to control) and relatively less important than for other progenitor cells in males (decrease of 84%). Consistent with this, it has been reported that the MPP2 population is heterogeneous and may contain stem cell activity [45]. Overall, in sharp contrast with results in males, chronotoxicity rhythms with moderate-to-large amplitudes were found in all studied outputs in female mice treated at 5 mg/kg. The acrophases of all investigated variables almost aligned in time since respective best timing according to BWL, circulating WBC, bone marrow cellularity and progenitor counts occurred at ZT15, ZT21, ZT15h15 and ZT14h45.

Next, low BWL and WBC toxicity in male mice treated with 5 mg/kg of oxaliplatin led us to test the dose of 10 mg/kg in these animals to investigate the appearance of possible timing dependencies. Indeed, BWL was increased in a dose-dependent manner and was three-fold greater at 10 mg/kg than at 5 mg/kg. Moreover, a significant circadian rhythm was validated with the best timing of oxaliplatin administration at ZT11h41 ± 48 min. Increasing the dose to 10 mg/kg in males increased the toxicity on WBC and led to a chronotoxicity rhythm with best timing at ZT15h06 ± 1 h 15, which was consistent with that of BWL. These results were further consistent with previous studies by Boughattas et al., who found a minimum of BWL at ZT5 ± 5 h and a maximum of WBC toxicity around ZT19 in B6D2F1 male mice treated with oxaliplatin (17 mg/kg) [31]. As expected, the 10 mg/kg injection further increased the toxicity on bone marrow cellularity and immature cell death as compared to the 5 mg/kg dose and no chronotoxicity variations could be observed. 

To some extent, chronotoxicity rhythms obtained in males treated with 10 mg/kg of oxaliplatin shared some similarities with that of females administered with 5 mk/kg. Although BWL mesors were different between both conditions (−0.44 ± 0.6% for the females treated at 5 mg/kg vs. −4.02 ± 0.41% for males treated with 10 mg/kg), the absolute amplitudes of BWL rhythms were close and were, respectively, 2.58 ± 0.64% for males (10 mg/kg) and 2.88 ± 3.89% for females (5 mg/kg). Best times of oxaliplatin administration with respect to BWL were ZT11h41 ± 48 min for males (10 mg/kg) and ZT13h59 ± 1 h 10 for females (5 mg/kg) so that a shift of +2 h 20 min between males and females was observed on average. In addition, the doses of 5 mg/kg in females and 10 mg/kg in males were equitoxic for both total WBC and lymphocyte counts, as similar mesors and amplitudes were found (Table 1 and Table S1). However, best timing was different and equal to ZT15h06 ± 1 h 15 min for males (10 mg/kg) and ZT21h02 ±1 h 31 for females (5 mg/kg) so that, as for BWL, a shift of +5 h 56 min between males and females was observed on average. For bone marrow cellularity, the doses of 5 mg/kg in females and 10 mg/kg in males were not equitoxic, as males displayed more toxicity. Overall, these findings suggest a phase shift between +2 h and +6 h between optimal timing for male and female mice.

Overall, sex strongly influenced oxaliplatin chronotoxicity profiles, with higher amplitudes in females and optimal timing being earlier in male mice than in female mice. Interestingly, in the same mouse strain, a male-to-female shift of +1 h 15 was found in BWL induced by the anticancer drug irinotecan and circadian amplitudes were larger in females as compared to males [26]. Similarly, in the clinics, a men-to-women shift of +6 h and larger amplitudes in women were observed regarding irinotecan best timing regarding both clinical and hematological toxicities, when administered in combination with 5-FU and oxaliplatin to colorectal cancer patients [29]. These results suggest that women may benefit more from proper drug timing as compared to men, since they display greater amplitudes in their circadian physiology and their chronotoxicity rhythms. All in all, this study underlines the need for the development of adapted chronotherapeutics protocols differently in men and women, in order to achieve the best outcomes.

In conclusion, this study provided insights into the sex dependency of the steady-state circadian physiology and oxaliplatin chronotoxicity of hematopoietic components. In control conditions, although circadian rhythms of circulating blood cell counts were similar between sexes, the underlying bone marrow cell dynamics presented higher amplitudes in females and a male-to-female phase shift of more than a quarter of the 24 h period. Further, BWL, blood cell counts, bone marrow cellularity and seven flow cytometry-monitored hematopoietic progenitor populations were evaluated 72 h after oxaliplatin injection. In females treated at 5 mg/kg, almost aligned chronotoxicity rhythms were found for all measured variables. In males treated at the same dose, almost no endpoints showed circadian rhythms, BWL and WBC toxicity being minimal, albeit with a substantial drop in BM progenitor counts. Increasing the dose to 10 mg/kg in males induced circadian rhythms in BWL and WBC but not in BM endpoints. Our results suggest complex and sex-specific clock-regulated mechanisms governing the response to oxaliplatin.

## Figures and Tables

**Table 1:** Cosinor parameters related to WBC, BM cellularity and progenitor counts, in physiological and treated conditions.

		Female Control	Male Control		Female 5 mg/kg	Male 5 mg/kg	Male 10 mg/kg
**WBC**	**Mesor (10^9^ cell/L)**	48.07 ± 1.82	45.12 ± 1.86	**Mesor (% of control)**	66.65 ± 6.16	79.40 ± 15.59	61.44 ± 5.58
	**Ampl. (% of Mesor)**	51.08 ± 5.485.79	51.97 ± 6.14	**Ampl. (% of Mesor)**	32.74 ± 13.42	NS	38.87± 13.58
	**Phase (ZT)**	5 h 42 ± 24 min	5 h 21 ± 26 min	**Phase (ZT)**	21 h 02 ±1 h 31	NS	15 h 06 ± 1 h 15
	***p*** **values**	<0.001	<0.001	***p*** **values**	0.049	0.75	0.016
**BM cellularity**	**Mesor (10^7^ cell/L)**	3.28 ± 0.14	3.47 ± 0.12	**Mesor (% of control)**	41.81 ± 1.22	40.58 ± 0.91	35.52 ± 0.88
	**Ampl. (% of Mesor)**	17.06 ± 6.14	12.35 ± 4.85	**Ampl. (% of Mesor)**	18.87 ± 4.15	8.36 ± 3.19	NS
	**Phase (ZT)**	13 h 42 ± 1 h 22	20 h 10 ± 1 h 28	**Phase (ZT)**	15 h 19 ± 51 min	16 h 27 ± 1 h 26	NS
	***p*** **values**	0.027	0.045	***p*** **values**	<0.001	0.04	0.26
**LK**	**Mesor (10^5^ cell/L)**	3.18 ± 0.17	2.26 ± 0.56	**Mesor (% of control)**	29.22 ± 1.46	1.59 ± 0.001	0.44 ± 0.0001
	**Ampl. (% of Mesor)**	38.84 ± 7.44	NS	**Ampl. (% of Mesor)**	27.55 ± 7.37	NS	NS
	**Phase (ZT)**	3 h 22 ± 47 min	NS	**Phase (ZT)**	15 h 27 ± 59 min	NS	NS
	***p*** **values**	<0.001	0.51	***p*** **values**	0.002	0.89	0.22
**LKS**	**Mesor (10^4^ cell/L)**	7.56 ± 0.43	5.96 ± 0.19	**Mesor (% of control)**	35.07 ± 1.53	2.67 ± 0.008	1.43 ± 0.002
	**Ampl. (% of Mesor)**	40.23 ± 8.09	14.35 ± 4.53	**Ampl. (% of Mesor)**	35.21 ± 6.47	NS	NS
	**Phase (ZT)**	3 h 20 ± 49 min	21 h 39 ± 1 h 14	**Phase (ZT)**	14 h30 ± 40 min	NS	NS
	***p*** **values**	<0.001	0.009	***p*** **values**	<0.001	0.27	0.32
**MPP1**	**Mesor (10^4^ cell/L)**	4.32 ± 0.26	3.77 ± 0.14	**Mesor (% of control)**	24.31 ± 0.83	1.25 ± 0.002	1.02 ± 0.002
	**Ampl. (% of Mesor)**	43.72 ± 8.68	NS	**Ampl. (% of Mesor)**	21.62 ± 4.92	NS	NS
	**Phase (ZT)**	3 h 15 ± 48 min	NS	**Phase (ZT)**	14 h 10 ± 51 min	NS	NS
	***p*** **values**	<0.001	0.077	***p*** **values**	<0.001	0.35	0.37
**MPP2**	**Mesor (10^3^ cell/L)**	6.24 ± 0.68	4.78 ± 0.31	**Mesor (% of control)**	80.15 ± 23.51	7.63 ± 0.08	2.24 ± 0.007
	**Ampl. (% of Mesor)**	52.17 ± 15.15	NS	**Ampl. (% of Mesor)**	229.67 ± 80.87	NS	NS
	**Phase (ZT)**	2 h 16 ± 1 h 09	NS	**Phase (ZT)**	13 h 52 ± 41 min	NS	NS
	***p*** **values**	0.003	0.12	***p*** **values**	<0.001	0.24	0.80
**ST-HSC**	**Mesor (10^4^ cell/L)**	1.22 ± 0.06	0.87 ± 0.041	**Mesor (% of contro)**	23.30 ± 0.82	1.68 ± 0.003	1.74 ± 0.005
	**Ampl. (% of Mesor)**	25.95 ± 7.61	22.38 ± 6.89	**Ampl. (% of Mesor)**	20.59 ± 5.11	NS	NS
	**Phase (ZT)**	4 h 22 ± 1 h 11	19 h 53 ± 1 h 09	**Phase (ZT)**	14 h 27 ± 55 min	NS	NS
	***p*** **values**	0.005	0.007	***p*** **values**	<0.001	0.29	0.14
**HSC-SLAM**	**Mesor (10^4^ cell/L)**	1.32 ±0.11	0.82 ± 0.028	**Mesor (% of control)**	61.87 ± 8.23	7.77 ± 0.06	2.53 ± 0.006
	**Ampl. (% of Mesor)**	37.89 ± 11.28	18.24 ± 4.93	**Ampl. (% of Mesor)**	103.38 ± 23.88	NS	NS
	**Phase (ZT)**	3 h 37 ± 1 h 13	19 h 40 ± 1 h 01	**Phase (ZT)**	14 h57 ± 41 min	NS	NS
	***p*** **values**	0.005	0.002	***p*** **values**	<0.001	0.062	0.70
**LT-HSC**	**Mesor (10^3^ cell/L)**	4.91± 0.25	3.05 ± 0.14	**Mesor (% of control)**	33.13 ± 2.10	1.89 ± 0.004	2.16 ± 0.005
	**Ampl. (% of Mesor)**	42.08 ± 7.35	NS	**Ampl. (% of Mesor)**	45.03 ± 9.66	0.8 ± 0.3	NS
	**Phase (ZT)**	4 h 09 ± 42 min	NS	**Phase (ZT)**	15 h 28 ± 45 min	8 h 16 ± 1 h 28	NS
	***p*** **values**	<0.001	0.58	***p*** **values**	<0.001	0.04	0.06

## Data Availability

Datasets will be made available at publication.

## Supplementary Materials

The following supporting information can be downloaded at: https://www.mdpi.com/article/10.3390/pharmaceutics14112465/s1, Figure S1: Sex- and timing-dependent circulating lymphocyte, monocyte and neutrophil counts; Figure S2: Sex- and timing-dependent red blood cell and platelet counts; Figure S3: Timing-dependent bone marrow progenitor cell numbers for the seven studied populations in males treated with 5 and 10 mg/kg; Table S1: Cosinor parameters related to circadian rhythms of circulating lymphocytes, monocytes, and neutrophils.
