# Supplementary material for: Sex and Circadian Timing Modulate Oxaliplatin Hematological and Hematopoietic Toxicities
Source: Pharmaceutics. 2022 Nov 15;14(11):2465. doi: 10.3390/pharmaceutics14112465 (PMC9699532; doi:10.3390/pharmaceutics14112465)
Supplement: Supplementary file 1 [file pharmaceutics-14-02465-s001.zip › pharmaceutics-1881263-supplementary.pdf]

SUPPLEMENTARY MATERIALS

		Female Control	Male Control		Female 5mg/kg	Male 5mg/kg	Male 10mg/kg
Lymphocytes	Mesor (10^9 cell/L)	42.58±1.68	37.62±1.66	Mesor (% of control)	62.64±5.63	79.08±15.73	54.67±3.74
	Ampl. (% of Mesor)	51.9± 6.09	51.8 ± 6.57	Ampl. (% of Mesor)	34.17 ± 11.69	NS	24.67 ± 9.98
	Phase (ZT)	6h ± 25min	5h19 ± 28 min	Phase (ZT)	22h07± 1h16	NS	14h27 ± 1h29
	p values	<0.001	<0.001	p values	0.014	0.60	0.048
Monocytes	Mesor (10^9 cell/L)	0.99±0.07	1.52±0.16	Mesor (% of control)	86.21±63.73	98.68±798	94.90±151.79
	Ampl. (% of Mesor)	29.36±10.9	45.8±15.6	Ampl. (% of Mesor)	NS	NS	NS
	Phase (ZT)	6h56 ± 6min	4h27±1h14	Phase (ZT)	NS	NS	NS
	p values	0.026	0.012	p values	0.66	0.92	0.29
Neutrophils	Mesor (10^9 cell/L)	4.48±0.31	6.45±0.44	Mesor (% of control)	118.43±102	71.43±21.14	88.35±68.81
	Ampl. (% of Mesor)	64.43± 10.77	51.83 ± 10.26	Ampl. (% of Mesor)	NS	NS	50.9± 42.7
	Phase (ZT)	3h07 ± 35 min	4h25 ± 43 min	Phase (ZT)	NS	NS	14h53± 1h04
	p values	<0.001	<0.001	p values	0.5	0.14	0.045

Table S1. Cosinor parameters related to circadian rhythms of circulating lymphocytes, monocytes and neutrophils

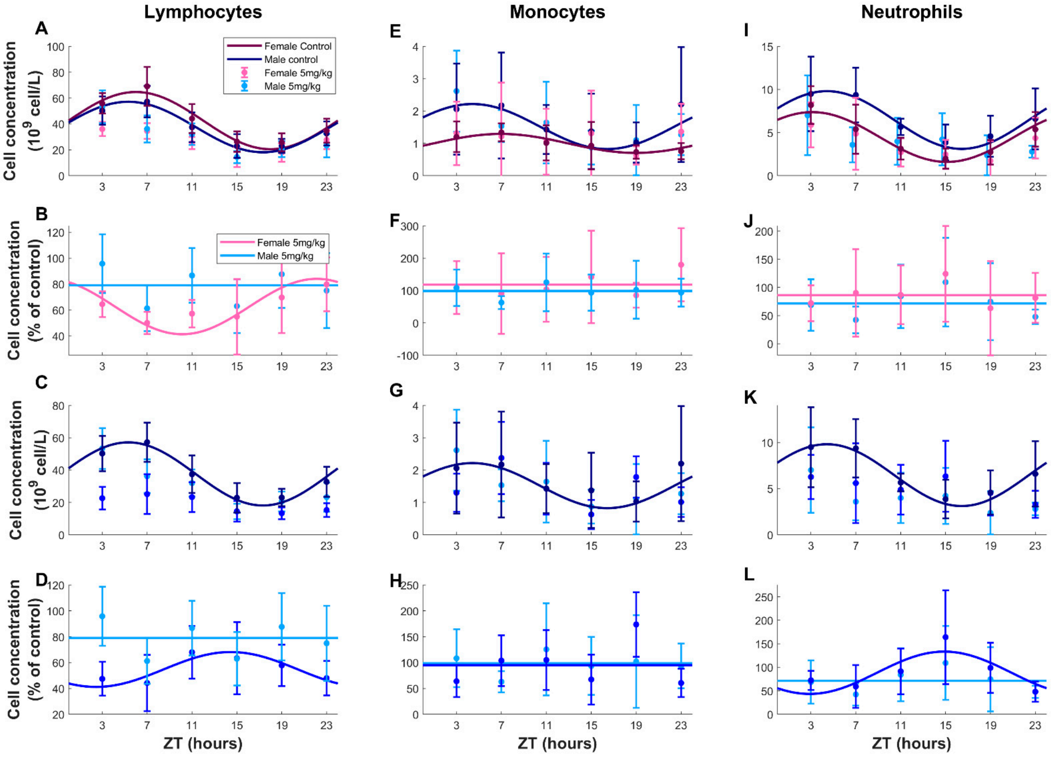

Figure S1. Sex- and timing-dependent circulating lymphocyte, monocyte and neutrophil counts: lymphocytes (**A-D**), monocytes (**E-H**), and neutrophils (**I-L**) platelets. Panels A, C, E, G, I, K show cell concentrations in control (datapoints, mean +/-SD) and treated (bars) conditions**.** Panels B, D, F, H, J, L show ratios between treated and control conditions (datapoints, mean +/-SD). On all panels, solid lines are the best-fit significant cosine curves. Data for males treated at 5mk/kg are repeated to ease comparison.

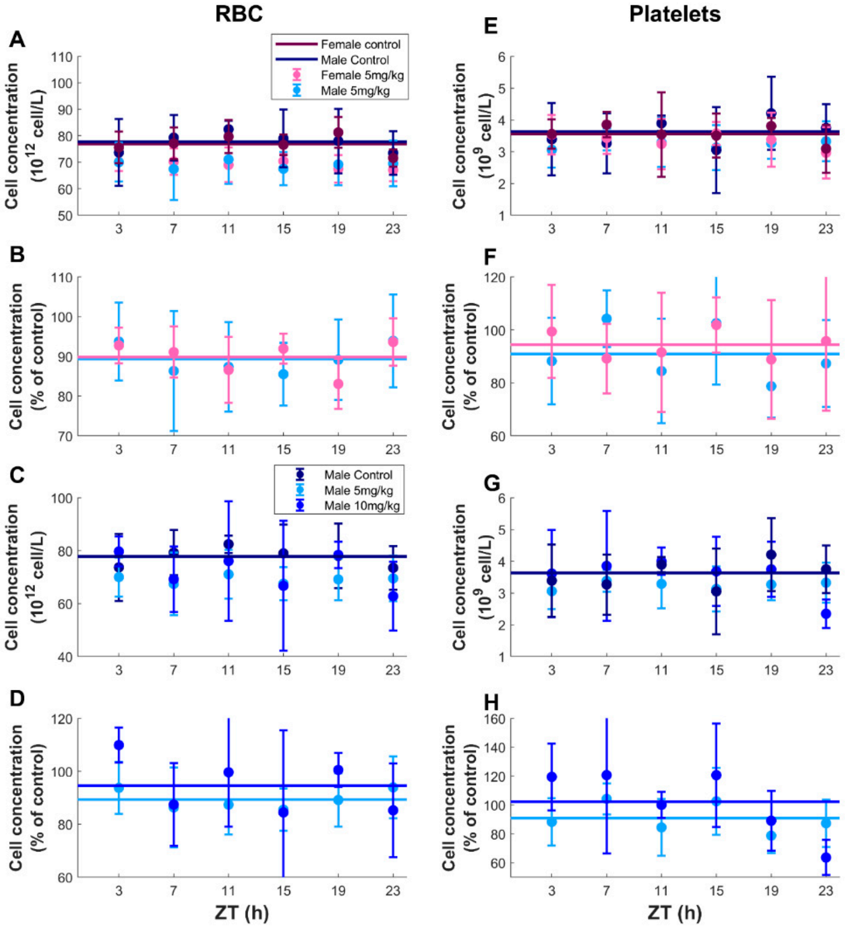

Figure S2. Sex- and timing -dependent red blood cell and platelet counts (**A,B,C,D**) Red blood cells, (**E,F,G,H**) Platelets. Panels A,C,E,G show cell numbers in control and treated conditions (datapoints, mean +/-SD). Panels B, D, F, H show ratios between treated and control conditions (datapoints, mean +/-SD). On all panels, solid lines are the best-fit significant cosine curves.

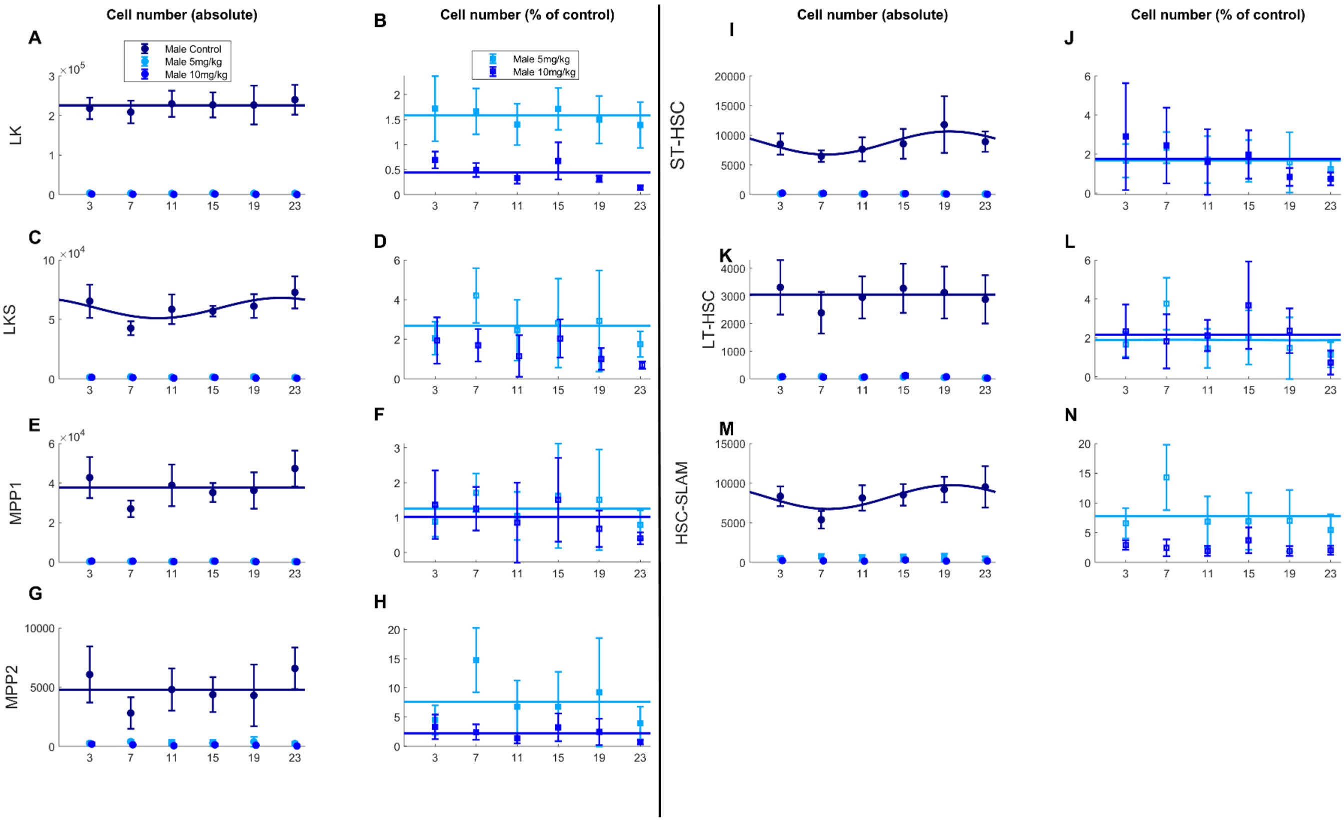

Figure S3. Timing-dependent bone marrow progenitor cell numbers for the seven studied populations in males treated with 5 and 10 mg/kg. Panels A, C, E, G, I, K, M show cell numbers in control and treated conditions (datapoints, mean +/-SD)**.** Panels B, D, F, H, J, L, N show ratios between treated and control conditions (datapoints, mean +/-SD). On all panels, solid lines are the best-fit significant cosine curves.
